# Development of Three Multiplex PCR Assays Targeting the 21 Most Clinically Relevant Serogroups Associated with Shiga Toxin-Producing *E. coli* Infection in Humans

**DOI:** 10.1371/journal.pone.0117660

**Published:** 2015-01-28

**Authors:** Sergio Sánchez, María Teresa Llorente, María Aurora Echeita, Silvia Herrera-León

**Affiliations:** Laboratory of Enterobacteriaceae, Service of Bacteriology, National Center of Microbiology, Institute of Health Carlos III, Majadahonda, Madrid, Spain; USDA-ARS-ERRC, UNITED STATES

## Abstract

*Escherichia coli* serogroups O5, O15, O26, O45, O55, O76, O91, O103, O104, O111, O113, O118, O121, O123, O128, O145, O146, O157, O165, O172, and O177 are the O-antigen forms of the most clinically relevant Shiga toxin-producing *E. coli* (STEC) serotypes. In this study, three multiplex PCR assays able to specifically detect these 21 serogroups were developed and validated. For this purpose, the O-antigen gene clusters of *E. coli* O5 and O76 were fully sequenced, their associated genes were identified on the basis of homology, and serogroup-specific primers were designed. After preliminary evaluation, these two primer pairs were proven to be highly specific and suitable for the development of PCR assays for O5 and O76 serogroup identification. Specific primers were also designed for serogroups O15, O45, O55, O91, O104, O113, O118, O123, O128, O146, O157, O165, O172, and O177 based on previously published sequences, and previously published specific primers for serogroups O26, O103, O111, O121, and O145 were also included. These 21 primer pairs were shown to be specific for their target serogroup when tested against *E. coli* type strains representing 169 known O-antigen forms of *E. coli* and *Shigella* and therefore suitable for being used in PCR assays for serogroup identification. In order to validate the three multiplex PCR assays, 22 *E. coli* strains belonging to the 21 covered serogroups and 18 *E. coli* strains belonging to other serogroups were screened in a double-blind test and their sensitivity was determined as 1 ng chromosomal DNA. The PCR assays developed in this study could be a faster, simpler, and less expensive strategy for serotyping of the most clinically relevant STEC strains in both clinical microbiology and public health laboratories, and so their development could benefit for clinical diagnosis, epidemiological investigations, surveillance, and control of STEC infections.

## Introduction

Shiga toxin (Stx)-producing *Escherichia coli* (STEC) are important food-borne zoonotic pathogens responsible for a broad spectrum of clinical symptoms in humans, ranging from mild diarrhea to hemorrhagic colitis (HC) and the life-threatening hemolytic uremic syndrome (HUS) [1]. Although serotype O157:H7 has been implicated in most outbreaks and in most cases of HUS, there is growing concern about the risk to human health associated with non-O157 STEC serotypes [2,3], which may be as well responsible for important outbreaks, such as the renowned 2011 STEC O104:H4 German outbreak [4].

*E*. *coli* clones including both pathogenic and commensal ones are currently identified by the combination of their O (lipopolysaccharide) and H (flagellum protein) antigens [1]. To date, more than 200 different STEC O:H serotypes have been associated with human disease [5]. However, the majority of clinical STEC infections, particularly those associated with outbreaks and serious patient outcomes, are attributable to strains belonging to a subset of STEC serotypes called enterohemorrhagic *E*. *coli* (EHEC). This term was originally coined to denote strains that cause HC and HUS, produce Stx and attaching and effacing (A/E) lesions, and possess the 60-MDa virulence plasmid [6]. Concretely, STEC strains producing Stx and A/E lesions and possessing the 60-MDa plasmid are denoted as “typical EHEC”, which includes serotypes O157:H7, O26:H11, O103:H2, O111:H8, O121:H19, and O145:H28 [1,7]. Disease associated STEC strains that do not produce A/E lesions and/or do not possess the 60-MDa plasmid, less frequently involved in hemorrhagic diseases than typical EHEC but nonetheless a frequent cause of diarrhea, are denoted as “atypical EHEC”, which includes serotypes O91:H21, O113:H21, and O104:H21 [1,7,8], as well as O76:H19, O128:H2, O146:H28, and even O104:H4 [4,9,10]. Furthermore, in the recent years, new EHEC serotypes have emerged as an important cause of food-borne infections in humans, including serotypes O5:H-, O15:H2, O45:H2, O55:H7, O103:H25/H11, O118:H16, O123:H11, O165:H25, O172:H25, or O177:H-, which have been denoted as “emerging EHEC” [7,11–13].

*E*. *coli* serotyping is typically performed by agglutination reactions using antisera raised in rabbits against the different O and H standard reference strains [14]. However, traditional serotyping is both laborious and time consuming and it often generates equivocal results due to cross-reaction between different serogroups, and even no results when testing rough strains, which are refractory to typing. The reference technique requires the previous thermal inactivation, at different temperatures, of the capsule in order to expose the O antigens, and also the use of a wide collection of antisera, which is too costly for most laboratories and can only be generated by specialized laboratories with animal facilities. Thus, rapid, less expensive, and more specific molecular methods for identifying different *E*. *coli* serotypes are strongly needed.

Much of the O-antigen variation in *E*. *coli* is a consequence of the extensive genetic diversity within the *rfb* (O-antigen) gene cluster, which encodes many of the enzymes involved in O-antigen biosynthesis and assembly [15]. The *rfb* region maps flanked by the two housekeeping genes *galF* and *gnd* on the *E*. *coli* chromosome. Indeed, the JUMPstart sequence, which is a 39-bp conserved element located in the intergenic region between *galF* and the O-antigen gene cluster, and the *gnd* sequence, which is present downstream the cluster, have been used to successfully amplify the entire O157 O-antigen gene cluster by PCR [16]. The cluster typically includes three different types of genes: (i) genes encoding enzymes involved in the synthesis of the sugars that form the O subunit; (ii) genes encoding transferases, which assemble sugar substituents into the O subunit; and (iii) genes encoding proteins involved in processing and assembly steps to build the O antigen from the O subunit, such as *wzx* (enconding the O-antigen transporter or flippase) and *wzy* (enconding the O-antigen polymerase) [17]. Several genes in the O-antigen gene cluster, in particular *wzx* and *wzy*, show relatively low similarity among different *E*. *coli* serogroups, and therefore primers targeting *wzx* and *wzy* are generally used to develop serogroup-specific PCR assays [18–21].

In this study, the O-antigen gene clusters of *E*. *coli* O5 and O76 reference strains were fully sequenced, their associated genes were identified on the basis of homology, and specific primers targeting *wzx* were designed for each serogroup. Specific primers targeting *wzx* or *wzy* were also designed for serogroups O15, O45, O55, O91, O104, O113, O118, O123, O128, O146, O157, O165, O172, and O177 based on previously published sequences from each serogroup. Previously published specific primers targeting *wzx* of serogroups O26, O103, O111, O121, and O145 were added to the designed primers to further develop three serogroup-specific multiplex PCR assays able to detect these 21 serogroups. These PCR assays were shown to be highly specific and sensitive, and suitable for the detection of the most clinically relevant STEC serogroups.

## Materials and Methods

### Bacterial strains

Reference strains for *E*. *coli* O5 and *E*. *coli* O76 from Statens Serum Institute (SSI, Copenhagen, Denmark) were used for nucleotide sequence analysis of their O-antigen gene clusters. A collection of 10 *E*. *coli* O5 and 15 *E*. *coli* O76 strains isolated from feces of humans and animals and from food at different time periods in Spain [22–25] was used for the preliminary evaluation of the O5 and O76 serogroup-specific PCR primers designed (S1 Table). A collection of *E*. *coli* type strains representing 169 known O-antigen forms of *E*. *coli* and *Shigella* was used for testing of primer specificity and validation of the serogroup-specific multiplex PCR assays developed (S2 Table).

### Amplification, sequencing, and sequence analysis of the *E*. *coli* O5 and O76 O-antigen gene clusters

Chromosomal DNA from *E*. *coli* O5 and *E*. *coli* O76 reference strains was prepared with a QIAamp DNA Mini Kit (Qiagen, Valencia, CA, USA). Published oligonucleotides 482 (5′-CAC TGC CAT ACC GAC GAC GCC GAT CTG TTG CTT GG-3′) and 412 (5′-ATT GGT AGC TGT AAG CCA AGG GCG GTA GCG T-3′) complementary to the JUMPstart sequence and to the proximal end of *gnd* [11], respectively, were used to amplify their entire O-antigen gene clusters in a long PCR assay carried out with the Expand Long Template PCR System (Roche Diagnostics, Mannheim, Germany) in an ABI 2720 thermal cycler (Applied Biosystems, Foster City, CA, USA) as follows: denaturation at 94°C for 10 s, annealing at 64°C for 30 s, and extension at 68°C for 15 min, repeated ten times. For the next 20 cycles, the extension step was increased by 20 s each time. One initial denaturing step (94°C for 2 min) and one final elongation step (72°C for 7 min) were added. Sequencing of long PCR products was achieved by a primer-walking approach in an ABI 3730xl DNA Analyzer (Applied Biosystems). DNA sequence assembly and analysis, and primers design were performed with the Lasergene software 7.0 (DNAstar, Madison, WI, USA). Within the resulting sequences, putative coding regions were identified by using ORF Finder (http://www.ncbi.nlm.nih.gov/gorf/). BLAST and PSI-BLAST were used for searching databases, including GenBank, COG, and Pfam (http://www.ncbi.nlm.nih.gov/blast/).

### Evaluation of the *E*. *coli* O5 and O76 serogroup-specific PCR primers

Serogroup-specific primer pairs were designed based on the *wzx* sequences determined before from *E*. *coli* O5 and *E*. *coli* O76 reference strains (Table 1). In order to preliminarily evaluate the *E*. *coli* O5 and O76 serogroup-specific PCR primers designed, chromosomal DNA from 50 *E*. *coli* strains including 10 *E*. *coli* O5, 15 *E*. *coli* O76 (S1 Table), and 25 *E*. *coli* strains belonging to other serogroups were screened in a double-blind test. For DNA extraction, a 1-μl loop of bacterial growth was suspended in 0.5 ml of sterile distilled water, boiled for 5 min, and centrifuged at 10,000 rpm for 5 min. The supernatant was used directly as template DNA in the PCR assays, without previous dilution. Conventional PCR amplifying a single target gene was performed using DreamTaq DNA Polymerase (Thermo Fisher Scientific, Waltham, MA, USA), according to the manufacturer’s instructions, in an ABI 2720 thermal cycler (Applied Biosystems) as follows: denaturation at 94°C for 30 s, annealing at 58°C for 30 s, and extension at 72°C for 1 min, repeated 25 times. Each reaction contained 400–800 nM of each primer (Table 1) and 5 μl of template DNA in a final volume of 25 μl. Fragments were separated in 2% agarose (MS8 type, Pronadisa, Madrid, Spain) gels by unidirectional electrophoresis using TAE 1x buffer and visualized by staining with ethidium bromide. Fragment size was determined by comparison with 100 bp DNA ladders (Thermo Fisher Scientific).

**Table 1:** Primers and concentrations used in the serogroup-specific multiplex PCR assays.

Multiplex	O type	Gene^a^	Primer	nM	Oligonucleotide sequence (5′-3′)	PCR product (bp)	Reference
1	O5	Wzx	wzx5_F	800	CTTATCCGATTAATGGCTTC	132	This study
			wzx5_R	800	TAGTCGATTTGCTTTTATGG		This study
1	O91	Wzy	wzy91_F9	600	TTTTCTGGAATGCTTGATGA	188	This study
			wzy91_R5	600	ATAATTTTACGCCGTGTTTG		This study
1	O26	Wzx	5′O26	400	ACTCTTGCTTCGCCTGTT	268	[18]
			3′O26	400	CAGCGATACTTTGAACCTTAT		[18]
1	O103	wzx	5′O103_F	400	TATCCTTCATAGCCTGTTGTT	327	[18]
			wzx103_R1	400	TTATAATAGTAATAAGCCAGACACC		This study
1	O145	wzx	5′O145.6	400	TTGAGCACTTATCACAAGAGATT	418	[18]
			3′O145.B	400	GATTGAATAGCTGAAGTCATACTAAC		[18]
1	O121	wzx	5′O121	200	GTAGCGAAAGGTTAGACTGG	651	[18]
			3′O121	200	ATGGGAAAGCTGATACTGC		[18]
1	O111	wzx	5′O111.3	400	GTTGCGAGGAATAATTCTTCA	829	[18]
			3′O111.2	400	CCATAGATATTGCATAAAGGC		[18]
2	O55	wzx	wzx55_F	200	ATCGCAATTGCAATAAACTC	144	This study
			wzx55_R1	200	CCCAACTCTAGTAGATAAAAGCC		This study
2	O128	wzx	wzx128_F2	200	TTTCGATCGTCTTGTTCAGG	193	This study
			wzx128_R1	200	CAATGGGCAATTAACACAGAG		This study
2	O113	wzy	wzy113_F1	400	TAACGGGATTAGAAGTGGAT	294	This study
			wzy113_R1	400	ATATAAGGCAGAAATGAGAGG		This study
2	O146	wzy	wzy146_F2	800	ATCAGTTCATGGGTTGTATTC	390	This study
			wzy146_R1	800	AGGAACATGGATGAAAGAAG		This study
2	O76	wzx	wzx76_F4	400	CATATGCAGATTGAAGGTAG	550	This study
			wzx76_R5	400	GAAAGCCATAAAGTGCC		This study
2	O45	wzx	wzx45_F	200	GACTTTCGTTGCGTTGTG	608	This study
			wzx45_R1	200	CTGCAAGTGTAGCGAAAAC		This study
2	O177	wzx	wzx177_F2	400	TCGGTGTTTGAAGGGGAAG	767	This study
			wzx177_R2	400	GTCCATGCATATGCCGTTC		This study
3	O157	wzx	wzx157_F3	600	CTCAATTTATAAAAAAGACGCTC	111	This study
			wzx157_R1	600	TCCAAATATTAACGACTTCACTAC		This study
3	O15	wzx	wzx15_F1	200	GCGTTGCCTACTTACTTATTATC	225	This study
			wzx15_R2	200	ATGCAAGTCCAGCCAAAC		This study
3	O104^b^	wzx	wzx104_F	400	CGGTGTATTAAGAAGTGTTGTC	272	This study
			wzx104_F	400	ATACTCCCCATAGAAACGC		This study
3	O118^c^	wzx	wzx118_F	200	TGGAGAACAGATAGCAAGAGG	409	This study
			wzx118_R	200	TATCCGACAAACACGAACC		This study
3	O123	wzx	wzx123_F	200	GAAAGAACAGAATCAGACTATGC	510	This study
			wzx123_R	200	TGTGCTAGCGCTAAAGGAC		This study
3	O165	wzx	wzx165_F	200	AACTGTTTATCCGAAGTGGTAG	651	This study
			wzx165_R	200	CACGCTTTAACGCATACAG		This study
3	O172	wzx	wzx172_F	200	ATTGGGTAGCCTCAGTAAAG	823	This study
			wzx172_R	200	CAGTCCAAACAGTGACAGTATC		This study

^a^GenBank accession numbers for each of the gene targets: O5*wzx* (KM881565); O91*wzy* (AY035396); O103*wzx* (AP010958); O55*wzx* (NC_013941); O128*wzx* (AY217096); O113*wzx* (AF172324); O146*wzx* (DQ465249); O76*wzx* (KM881564); O45*wzx* (AY771223); O177*wzx* (DQ008593); O157*wzx* (AE005174); O15*wzx* (AY647261); O104*wzx* (AF361371); O118*wzx* (DQ990684); O123*wzx* (DQ676933); O165*wzx* (GU068045); O172*wzx* (AY545992).

^b^Cross reaction with *wzx* from *E*. *coli* O9.

^c^Cross reaction with *wzx* from *E*. *coli* O151.

### Specificity of the serogroup-specific PCR primers

Apart from the primers specific for *wzx* genes of *E*. *coli* O5 and O76 described before, specific primers targeting *wzx* or *wzy* were also designed for serogroups O15, O45, O55, O91, O104, O113, O118, O123, O128, O146, O157, O165, O172, and O177 based on previously published sequences from each serogroup (Table 1), and previously published specific primers targeting *wzx* of serogroups O26, O103, O111, O121, and O145 [18] (Table 1) were also considered to further develop multiplex PCR assays. The specificity of the 21 serogroup-specific PCR primer pairs was evaluated by testing them against 169 *E*. *coli* type strains representing a broad range of O antigens of *E*. *coli* and *Shigella*. For this purpose, chromosomal DNA was prepared as described before from each of the 169 type strains and used to make DNA pools. A total of 18 pools were made, each containing DNA from 4 to 10 strains (S2 Table), and the 21 serogroup-specific PCR primer pairs were used to screen the DNA pools. Conventional PCR was performed as described before, with each reaction containing 200–800 nM of each primer (Table 1).

### Validation and sensitivity of the serogroup-specific multiplex PCR assays

The 21 serogroup-specific primer pairs were combined in three multiplex 5′-nuclease PCR assays (multiplex 1 to 3) (Table 1). In order to validate the three multiplex PCR assays, 22 *E*. *coli* strains belonging to the 21 covered serogroups and 18 *E*. *coli* strains belonging to other serogroups selected from the *E*. *coli* type strains collection were screened in a double-blind test. For this purpose, chromosomal DNA from these 40 *E*. *coli* strains was prepared as described before and screened with the three multiplex PCR assays. PCR was performed as described before but in a multiplex way, with each multiplex reaction containing 7 primer pairs in total and 200–800 nM of each primer (Table 1). Additionally, the three multiplex PCR assays were used to test 10-fold serially diluted chromosomal DNA prepared from *E*. *coli* strains belonging to the 21 covered serogroups selected from the *E*. *coli* type strains collection.

### Nucleotide Sequence Accession Number

The DNA sequences of the *E*. *coli* O5 and O76 O-antigen gene clusters have been deposited in GenBank under the accession numbers KM881565 and KM881564, respectively.

## Results and Discussion

### Nucleotide sequence analysis of the *E*. *coli* O5 and O76 O-antigen gene clusters

**Fig 1:**
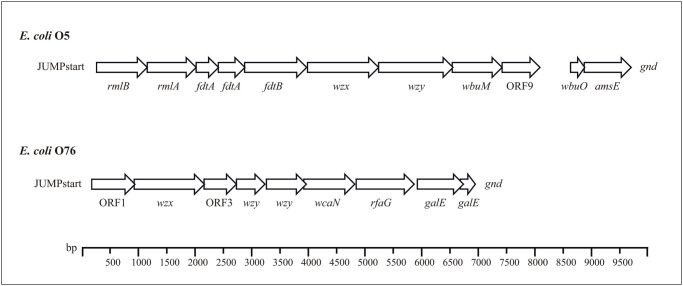
*E*. *coli* O5 and O76 O-antigen gene clusters. All genes are transcribed in a direction from the JUMPstart sequence to *gnd*.

A sequence of 9,915 bases between the JUMPstart sequence and *gnd* was obtained from *E*. *coli* O5, and eleven open reading frames (ORFs) were found (Fig. 1). All the ORFs were assigned functions and shown to be related to O-antigen biosynthesis on the basis of their similarity to related genes in nucleotide sequence databases (Table 2). A sequence of 7,134 bases was obtained from *E*. *coli* O76, and nine ORFs were found (Fig. 1). Likewise, all the ORFs were assigned functions and shown to be related to O-antigen biosynthesis on the basis of their similarity to related genes in nucleotide sequence databases (Table 3). As expected for O-antigen gene clusters, the sequences obtained had a significantly lower G+C content than those in the *E*. *coli* genome (Tables 2 and 3) [26]. The *E*. *coli* O5 O-antigen gene cluster was shown to be highly related to the *Salmonella* Pomona O28 O-antigen gene cluster (accession number EU805803), with the same organization and 78% DNA identity. *E*. *coli* and *Salmonella* are closely related, and several cases in which the O-antigen structures are identical or highly similar in the two species have been documented [27,28]. The sequence similarity level between *Salmonella* and *E*. *coli* O-antigen gene clusters that express identical O-antigen backbones is close to the lower end of the range for their housekeeping genes (between 76% and 100% DNA identity), indicating that O-antigen gene clusters for each structure originate from a common ancestor [28].

**Table 2:** Putative genes in the *E*. *coli* O5 O-antigen gene cluster.

Gene	Location in sequence	G+C content (%)	Similar protein(s), species (GenBank accession no.)	**% aa identity** ^a^	Putative function of protein
rmlB	260–1153	42.4	RmlB, *Escherichia coli* (WP_000699410)	100	dTDP-glucose 4,6-dehydratase
rmlA	1153–2016	38.4	RmlA, *Escherichia coli* (WP_000676087)	100	Glucose-1-phosphate thymidylyltransferase
fdtA	2020–2415	36.1	FdtA, *Escherichia coli* (WP_001025599)	100	dTDP-6-deoxy-3,4-keto-hexulose isomerase
fdtA	2412–2879	35.3	FdtA, *Escherichia coli* (WP_000469854)	100	dTDP-6-deoxy-3,4-keto-hexulose isomerase
fdtB	2876–3760	36.3	FdtB, *Escherichia coli* (WP_000564888)	99	Aminotransferase
wzx	3985–5241	31.9	Wzx, *Escherichia coli* (WP_001048967)	100	O-antigen flippase
wzy	5242–6558	31.7	Wzy, *Escherichia coli* (WP_000397255)	100	O-antigen polymerase
wbuM	6542–7420	31.4	WbuM, *Escherichia coli* (WP_001200008)	100	Glycosyltransferase
ORF9	7420–8088	30.8	Putative protein, *Escherichia coli* (WP_000472515)	100	Haloacid dehalogenase-like hydrolase
wbuO	8628–8879	30.2	WbuO, *Escherichia coli* (WP_001300987)	100	Serine transferase
amsE	8872–9699	33.9	AmsE, *Escherichia coli* (WP_001000076)	100	Amylovoran biosynthesis protein

^a^aa, amino acid.

**Table 3:** Putative genes in the *E*. *coli* O76 O-antigen gene cluster.

Gene	Location in sequence	G+C content (%)	Similar protein(s), species (GenBank accession no.)	**% aa identity** ^a^	Putative function of protein
ORF1	176–940	29.4	Putative protein, *Escherichia coli* (WP_024187517)	100	Glycosyltransferase
wzx	927–2165	30.1	Wzx, *Escherichia coli* (WP_001015334)	100	O-antigen flippase
ORF2	2162–2731	35.3	Putative protein LbH_MAT_like, *Escherichia coli* (WP_000759956)	100	Acetyltransferase
wzy	2731–3237	25.3	Wzy, *Escherichia coli* (WP_000005509)	100	O-antigen polymerase
wzy	3258–3962	30.6	Wzy, *Escherichia coli* (WP_000005509)	100	O-antigen polymerase
wcaN	3922–4821	31.8	WcaN, *Escherichia coli* (WP_000908761)	100	Glycosyltransferase
rfaG	4847–5872	32.8	RfaG, *Escherichia coli* (WP_000038788)	100	Glycosyltransferase
galE	5927–6739	34.9	GalE, *Escherichia coli* (WP_000699474)	100	UDP-glucose 4-epimerase
galE	6684–6950	33.4	GalE, *Escherichia coli* (WP_000699474)	100	UDP-glucose 4-epimerase

^a^ aa, amino acid.

### Evaluation of the *E*. *coli* O5 and O76 serogroup-specific PCR primers

Primer pairs specific for *wzx* genes of *E*. *coli* O5 and O76 were designed (Table 1) and evaluated in a double-blind test with 50 *E*. *coli* strains including 10 *E*. *coli* O5, 15 *E*. *coli* O76 (S1 Table), and 25 *E*. *coli* strains belonging to other serogroups. All the *E*. *coli* O5 and O76 strains gave the expected PCR products corresponding to primer pairs used (Table 1), and no PCR products were obtained from strains belonging to other serogroups. Thus, the primers were proven to be highly specific and suitable for the development of PCR assays for O5 and O76 serogroup identification.

### Identification of serogroup-specific genes by PCR

Primer pairs specific for *wzx* or *wzy* genes of *E*. *coli* O5, O15, O26, O45, O55, O76, O91, O103, O104, O111, O113, O118, O121, O123, O128, O145, O146, O157, O165, O172, and O177 (Table 1) were used to screen the 18 DNA pools containing representatives of 169 known O-antigen forms of *E*. *coli* and *Shigella* (S2 Table) in order to test their specificity. The pools containing strains of any of the 21 covered serogroups gave PCR products of the expected size (Table 1), and no PCR products were obtained from the remaining pools, with the only exceptions of pools 1 and 15. Pool 1 gave a positive PCR result when tested with the primer pair specific for *wzx* gene of *E*. *coli* O104 (272 bp). Such a result was not surprising, since pool 1 contained an *E*. *coli* O9 strain and it is well known that the gene cluster encoding for serogroup O104 has the same genes in the same order as the K9 gene cluster [29], and the K9 antigen is generally present in *E*. *coli* strains belonging to serogroups O8, O9, and O9a [30], which was exactly the case of the *E*. *coli* O9 strain included in pool 1. Likewise, pool 15 gave a positive result when tested with the primer pair specific for *wzx* gene of *E*. *coli* O118 (409 bp). This result was also expected, since pool 15 contained an *E*. *coli* O151 strain and the O-antigen gene clusters of both *E*. *coli* O118 and *E*. *coli* O151 have been shown to be organized in the same manner and to share high level identity (> 99% DNA identity) [28]. Indeed, the sequences of *wzx* genes of *E*. *coli* O118 and *E*. *coli* O151 are identical [28]. Thus, apart from these limitations, the 21 primer pairs were proven to be specific for their target serogroup when tested against *E*. *coli* type strains representing 169 different serogroups and therefore suitable for being used in PCR assays for serogroup identification.

### Development of serogroup-specific multiplex PCR assays

**Fig 2:**
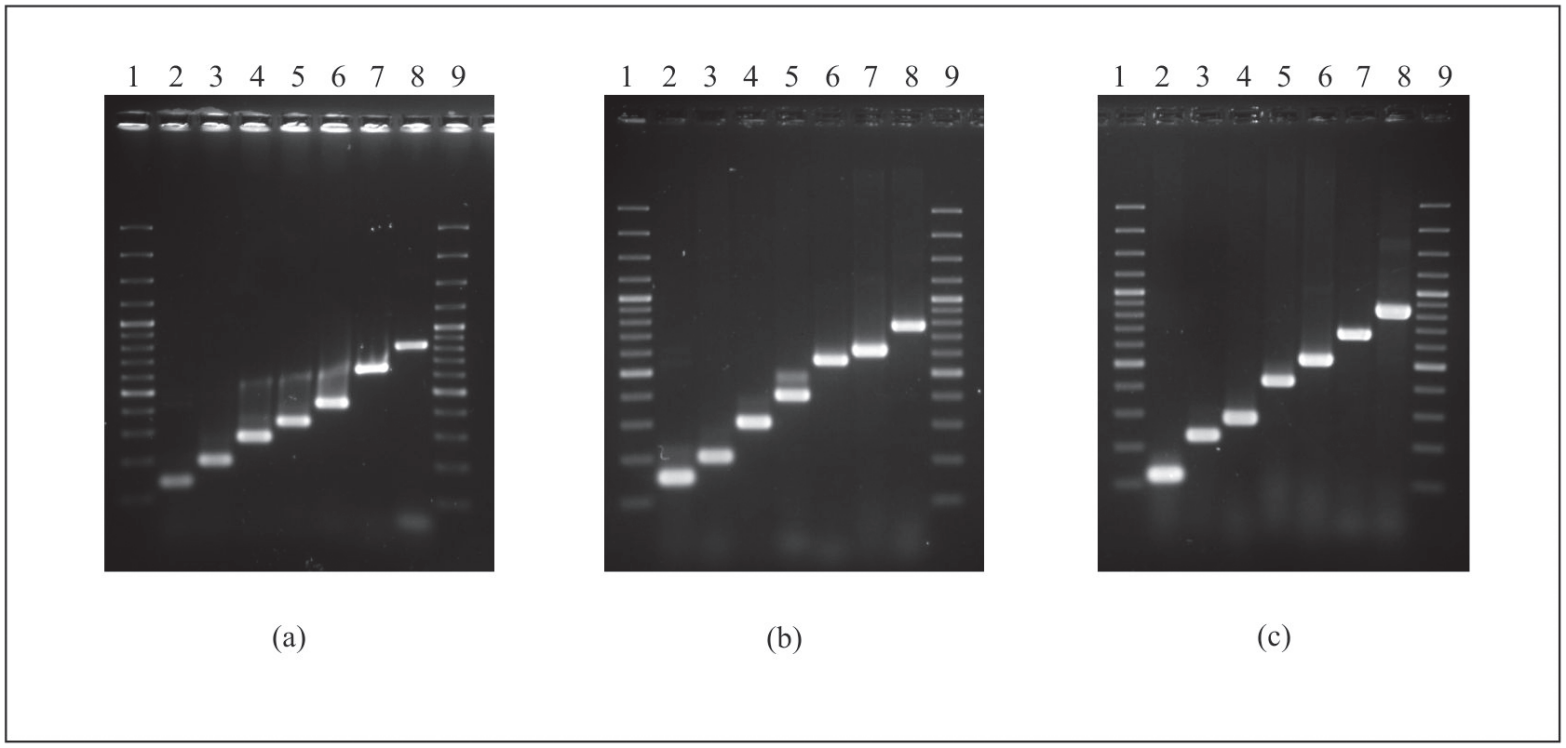
Agarose gel electrophoresis of the PCR products obtained from *E*. *coli* type strains belonging to the 21 covered serogroups by using the three multiplex PCR assays. **(a) Multiplex 1**: lanes 1 and 9, 100 bp DNA ladder; lane 2, O5; lane 3, O91; lane 4, O26; lane 5, O103; lane 6, O145; lane 7, O121; lane 8, O111. **(b) Multiplex 2**: lanes 1 and 9, 100 bp DNA ladder; lane 2, O55; lane 3, O128; lane 4, O113; lane 5, O146; lane 6, O76; lane 7, O45; lane 8, O177. **(c) Multiplex 3**: lanes 1 and 9, 100 bp DNA ladder; lane 2, O157; lane 3, O15; lane 4, O104; lane 5, O118; lane 6, O123; lane 7, O165; lane 8, O172.

In order to develop a less laborious PCR method, the 21 serogroup-specific primer pairs were combined in three multiplex 5′-nuclease PCR assays (multiplex 1 to 3) (Table 1) aiming to detect the most clinically relevant STEC serogroups. For this purpose, the primer pair efficiency for the 21 serogroups was determined on the basis of the amplicons of expected sizes by testing different primer concentrations. The primer concentration resulting in high-signal products was used as described in the Methods section. At the optimized primer concentration ratio, the DNA of 22 strains belonging to the 21 covered serogroups produced the expected PCR products (Fig. 2) in the double-blind test for validation of the three multiplex PCR assays. DNA from 17 strains belonging to other serogroups did not produce any other PCR products and one *E*. *coli* O9 strain gave a positive PCR result (272 bp) when tested with multiplex 3, due to the presence of the primer pair specific for *wzx* gene of *E*. *coli* O104 on this multiplex PCR assay, as discussed before. To test the sensitivity of the three multiplex PCR assays, they were carried out to amplify serially diluted chromosomal DNA (100 ng, 10 ng, 1 ng, 100 pg, 10 pg, and 1 pg) prepared from strains belonging to the 21 covered serogroups, and positive PCR results were obtained from as little as 1 ng of DNA for each of the strains.

In conclusion, the three serogroup-specific multiplex PCR assays developed in this study were found to be highly specific and sensitive, and suitable for serogroup identification in *E*. *coli*. The combination of these three multiplex PCR assays enables the reliable detection of genes encoding the O antigen in *E*. *coli* strains belonging to the most clinically relevant STEC serotypes, including typical, atypical, and emerging EHEC serotypes. This method of molecular serotyping is a faster, simpler, and less expensive technique than traditional serotyping, also enabling the detection of *E*. *coli* O antigens even when they cannot be expressed by the bacteria. As a consequence, these PCR assays could be an efficient and convenient strategy for serotyping of the most clinically relevant STEC strains in both clinical microbiology and public health laboratories, especially in those where PCR is already a routine tool, and so their development could benefit clinical diagnosis, epidemiological investigation, surveillance, and control of STEC infections.

## Supporting Information

S1 Table *E*. *coli* O5 and O76 strains used in this study. (DOCX)

S2 Table *E*. *coli* strains in the pools used for testing of primer specificity. (DOCX)
