# Supplementary material for: Development of Three Multiplex PCR Assays Targeting the 21 Most Clinically Relevant Serogroups Associated with Shiga Toxin-Producing E. coli Infection in Humans
Source: PLoS One. 2015 Jan 28;10(1):e0117660. doi: 10.1371/journal.pone.0117660 (PMC4309606; doi:10.1371/journal.pone.0117660)
Supplement: S1 Table — (DOCX) [file pone.0117660.s001.docx]

**Table S1. *E. coli* O5 and O76 strains used in this study.**

| **Strain** | **Serogroup** | **Source (yr of isolation)** | **Origin^a^** | **Reference** |
| --- | --- | --- | --- | --- |
| FCVTEC31 | O5 | Sheep (2004) | FV-UEX | [25] |
| FCVTEC64 | O5 | Sheep (2003) | FV-UEX | [25] |
| FCVTEC68 | O5 | Sheep (2004) | FV-UEX | [25] |
| FCVTEC89 | O5 | Sheep (2003) | FV-UEX | [25] |
| FCVTEC90 | O5 | Sheep (2004) | FV-UEX | [25] |
| FCVTEC139 | O5 | Sheep (2003) | FV-UEX | [25] |
| FCVTEC146 | O5 | Sheep (2004) | FV-UEX | [25] |
| FCVTEC148 | O5 | Sheep (2004) | FV-UEX | [25] |
| FCVTEC150 | O5 | Sheep (2004) | FV-UEX | [25] |
| FCVTEC208 | O5 | Sheep (2004) | FV-UEX | [25] |
| CNM-1482/12 | O76 | Human (2012) | CNM-ISCIII | [24] |
| CNM-2378/12 | O76 | Human (2012) | CNM-ISCIII | [24] |
| FVCC423 | O76 | Raw goat’s milk (2004) | FV-UEX | [23] |
| FCVTEC166 | O76 | Sheep (2003) | FV-UEX | [25] |
| FCVTEC167 | O76 | Sheep (2004) | FV-UEX | [25] |
| FCVTEC168 | O76 | Sheep (2003) | FV-UEX | [25] |
| FCVTEC169 | O76 | Sheep (2003) | FV-UEX | [25] |
| FCVTEC170 | O76 | Sheep (2003) | FV-UEX | [25] |
| FCVTEC171 | O76 | Sheep (2004) | FV-UEX | [25] |
| FCVTEC172 | O76 | Sheep (2003) | FV-UEX | [25] |
| FCVTEC174 | O76 | Sheep (2004) | FV-UEX | [25] |
| FCVTEC175 | O76 | Sheep (2004) | FV-UEX | [25] |
| FCVTEC205 | O76 | Sheep (2004) | FV-UEX | [25] |
| FCVTEC207 | O76 | Sheep (2004) | FV-UEX | [25] |
| IREC6 | O76 | Deer (2009) | IREC | [22] |

^a^FV-UEX, Faculty of Veterinary Sciences, University of Extremadura, Cáceres, Spain ; CNM-ISCIII, National Center of Microbiology, Institute of Health Carlos III, Majadahonda, Spain ; IREC, National Wildlife Research Institute, Spanish National Research Council/University of Castilla-La Mancha, Ciudad Real, Spain.
