# Supplementary material for: Development of Three Multiplex PCR Assays Targeting the 21 Most Clinically Relevant Serogroups Associated with Shiga Toxin-Producing E. coli Infection in Humans
Source: PLoS One. 2015 Jan 28;10(1):e0117660. doi: 10.1371/journal.pone.0117660 (PMC4309606; doi:10.1371/journal.pone.0117660)
Supplement: S2 Table — (DOCX) [file pone.0117660.s002.docx]

**Table S2. *E. coli* strains in the pools used for testing of primer specificity.**

| **Pool no.** | **Strains included in the pool** | **Origin^a^** |
| --- | --- | --- |
| 1 | *E. coli* strains for serogroups O1, O2, O3, O4, O5, O6, O7, O8, O9, O10 | FV-UEX |
| 2 | *E. coli* strains for serogroups O11, O12, O13, O14, O15, O16, O17, O18, O19, O20 | FV-UEX |
| 3 | *E. coli* strains for serogroups O21, O22, O23, O24, O25, O26, O27, O28, O29, O30 | FV-UEX |
| 4 | *E. coli* strains for serogroups O32, O33, O34, O35, O36, O37, O38, O39, O40, O41 | FV-UEX |
| 5 | *E. coli* strains for serogroups O42, O43, O44, O45, O46, O48, O49, O50, O51, O52 | FV-UEX |
| 6 | *E. coli* strains for serogroups O53, O54, O55, O56, O57, O58, O59, O60, O61, O62 | FV-UEX |
| 7 | *E. coli* strains for serogroups O63, O64, O65, O66, O68, O69, O70, O71, O73, O74 | FV-UEX |
| 8 | *E. coli* strains for serogroups O75, O76, O77, O78, O79, O80, O81, O82, O83, O84 | FV-UEX |
| 9 | *E. coli* strains for serogroups O85, O86, O87, O88, O89, O90, O91, O92, O95 | FV-UEX |
| 10 | *E. coli* strains for serogroups O96, O97, O98, O99, O100, O101, O102, O103, O104, O105 | FV-UEX |
| 11 | *E. coli* strains for serogroups O106, O107, O108, O109, O110, O111, O112, O113 | FV-UEX |
| 12 | *E. coli* strains for serogroups O114, O115, O116, O117, O118, O119, O120, O121, O123, O124 | FV-UEX |
| 13 | *E. coli* strains for serogroups O125, O126, O127, O128, O129, O130, O131, O132 | FV-UEX |
| 14 | *E. coli* strains for serogroups O133, O134, O135, O136, O137, O138, O139, O140, O141, O142 | FV-UEX |
| 15 | *E. coli* strains for serogroups O143, O144, O145, O146, O147, O148, O149, O150, O151, O152 | FV-UEX |
| 16 | *E. coli* strains for serogroups O153, O154, O155, O156, O157, O158, O159, O160, O161, O162 | FV-UEX |
| 17 | *E. coli* strains for serogroups O163, O164, O165, O166, O167, O168, O169, O170, O171, O172 | FV-UEX |
| 18 | *E. coli* strains for serogroups O173, O174, O176, O177 | FV-UEX |

^a^FV-UEX, Faculty of Veterinary Sciences, University of Extremadura, Cáceres, Spain.
